# Transcriptome Analysis on Hepatopancreas Reveals the Metabolic Dysregulation Caused by *Vibrio parahaemolyticus* Infection in *Litopenaeus vannamei*

**DOI:** 10.3390/biology12030417

**Published:** 2023-03-09

**Authors:** Miao Miao, Shihao Li, Yuan Liu, Yang Yu, Fuhua Li

**Affiliations:** 1 CAS and Shandong Province Key Laboratory of Experimental Marine Biology, Institute of Oceanology, Chinese Academy of Sciences, Qingdao 266071, China; miaomiao@qdio.ac.cn (M.M.); liuyuan@qdio.ac.cn (Y.L.); yuyang@qdio.ac.cn (Y.Y.); fhli@qdio.ac.cn (F.L.); 2 Laboratory for Marine Biology and Biotechnology, Qingdao National Laboratory for Marine Science and Technology, Qingdao 266237, China; 3 University of Chinese Academy of Sciences, Beijing 100049, China; 4 Center for Ocean Mega-Science, Chinese Academy of Sciences, Qingdao 266071, China; 5 The Innovation of Seed Design, Chinese Academy of Sciences, Wuhan 430072, China

**Keywords:** transcriptomics, hepatopancreas, metabolism, *Vibrio* infection, shrimp

## Abstract

**Simple Summary:**

Acute hepatopancreas necrosis disease (AHPND) is a lethal disease which hinders the development of shrimp aquaculture. It is mainly caused by *Vibrio parahaemolyticus*. In order to learn more about the mechanism of resistance to AHPND and breed the disease resistant broodstocks, transcriptome analysis has been widely used to study the immune responses of shrimp to *Vibrio parahaemolyticus* infection, and many immune-related genes have been reported in response to the pathogen. However, few studies have focused on the relationship between the host metabolism and *Vibrio* infection. In this study, we performed a comparative transcriptomic analysis on the hepatopancreas of shrimp at different times after *V. parahaemolyticus* infection. We found that several processes and pathways related to metabolism were significantly upregulated in shrimp hepatopancreas after infection. These results indicate that the metabolism of shrimp plays an important role in response to *Vibrio* infection. The data provide a new perspective for the development of disease-resistant strategies in shrimp aquaculture.

**Abstract:**

Acute hepatopancreas necrosis disease (AHPND) has caused massive deaths of shrimp and has led to huge economic losses in aquaculture. *Vibrio parahaemolyticus* (VP_AHPND_) carrying a plasmid encoding binary toxins homologous to the *photorhabdus* insect-related (Pir) toxins is one of the main pathogens causing this disease. Previous studies have reported many immune-related genes of shrimp in response to this pathogenic bacteria. However, few studies have so far focused on the metabolic changes in *Litopenaeus vannamei* upon VP_AHPND_ infection. In the present study, comparative transcriptomic analysis was performed on the hepatopancreas of shrimp at different times during VP_AHPND_ infection. Functional analyses on the differentially expressed genes (DEGs) during infection showed that pathways related to glucose, energy and amino acid metabolism, as well as nucleic acid synthesis, were obviously changed in the hepatopancreas after VP_AHPND_ infection. Additionally, three signaling pathways, which could regulate metabolic processes, including HIF-1 signaling pathway, PI3K-Akt signaling pathway and NF-KappaB signaling pathway, also changed significantly. Collectively, these data reveal a close relationship between host metabolism processes and *Vibrio* infection. The information will enrich our understanding of the interaction mechanism between the shrimp and *Vibrio*.

## 1. Introduction

The Pacific white shrimp *Litopenaeus vannamei* is one of the major shrimp species cultivated around the world. According to the FAO, its production was estimated about 5812 thousand tons in 2020 [1]. However, shrimp diseases caused by bacteria, viruses and fungi threaten the development of the shrimp industry [2,3]. The emerging lethal disease named acute hepatopancreas necrosis disease (AHPND) has caused a tremendous decrease in shrimp production and has become one of the greatest hindrances to the development of shrimp aquaculture [4]. The causative agent of AHPND was originally reported to be a specific *Vibrio parahaemolyticus*, which carries an extrachromosomal plasmid that encodes a binary toxin *PirA^Vp^* and *PirB^Vp^*, homologous to the *Photorhabdus* insect-related (Pir) toxins [5]. Additionally, studies have demonstrated that other *Vibrio* species, such as *Vibrio owensii*, *Vibrio harveyi*, *Vibrio campbellii* and *Vibrio punensis*, which contain plasmid-coded toxin *PirAB^vp^*, could also result in AHPND [6,7,8,9]. Moreover, a study has also shown that the unfavorable aquatic conditions make farmed shrimp more susceptible to *Vibrio* [10].

The clinical symptoms of shrimp with AHPND include lethargy, bottom swimming, empty stomach and intestine, pale and atrophied hepatopancreas [4,11]. The hepatopancreas is the main target tissue of AHPND in shrimp. VP_AHPND_ infection could cause the sloughing and necrosis of epithelial cells in hepatopancreas tubules and massive hemocytic infiltration [12]. As a multifunctional organ, the hepatopancreas integrates metabolic and immune functions in crustaceans [13]. It not only participates in nutrient metabolism, but also in pathogen clearance and antigen processing [14]. In recent years, transcriptome analysis has been widely used to study the immune responses of shrimp to *V. parahaemolyticus* infection, and many immune-related genes in response to the pathogen have been found [15,16]. However, few studies have specifically focused on the metabolic changes in the shrimp hepatopancreas during *V. parahaemolyticus* infection.

In fact, the metabolic processes in aquatic animals play an important role in the pathogenesis of bacterial infections. For example, in the hemocytes of shrimp, white spot syndrome virus (WSSV) causes the Warburg effect of the host to meet its own demand for energy and macromolecular precursors [17]. Furthermore, transcriptome studies on different tissues of shrimp have also shown that pathogen infection causes metabolic changes in the host [18,19]. In addition, these changes are also found in other marine aquatic species, such as the Clam *Ruditapes philippinarum* [20]. Nevertheless, the detailed changes in shrimp metabolism caused by *V. parahaemolyticus* infection are still less investigated.

In the present study, we focused on the hepatopancreas of shrimp during *V. parahaemolyticus* infection and explored the details of metabolic changes in the tissue responsive to *V. parahaemolyticus* infection. The results provided us with a deeper understanding of the interaction between host and pathogen, and provided new ideas for the development of disease resistance methods from the perspective of metabolism.

## 2. Materials and Methods

### 2.1. Shrimp Culture and VP_AHPND_ Challenge

Healthy *L. vannamei* were provided by Hainan Grand Suntop Ocean Breeding Co., Ltd. (Wenchang, China) and cultured in filtered seawater at 25 °C and continuously aerated. The VP_AHPND_ were isolated from shrimp infected with *V. parahaemolyticus*. The procedure of isolation was as described by Liu et al. [21]. Firstly, the hepatopancreas of shrimp with AHPND was extracted to a centrifugal tube with sterile phosphate-buffered saline (PBS) and crushed into homogenates. The tissue homogenates were diluted and coated in citrate thiosulfate bile sucrose (TCBS) agar medium. After incubation at 28 °C for 18 h, monoclones were re-steaked on TCBS agar medium. The monoclone was selected again and inoculated into liquid tryptic soy broth (with 2% NaCl) medium. The cultured bacteria were centrifuged and boiled to extract DNA. Then, the DNA was used as a template to amplify 16s rRNA, *PirA^Vp^* and *PirB^Vp^* toxins. The products of 16s rRNA were sequenced and blasted in NCBI, the products of *PirA^Vp^* and *PirB^Vp^* toxins were detected by 1% agarose gel electrophoresis. The *Vibrio* used in the infection experiment was prepared as described by Zhang et al. [22]. Immersion infection was carried out to mimic the natural state of shrimp infection. Before the experiment, shrimp were bred in the aquarium at laboratory to acclimatize to the environment. About 100 healthy shrimp were used in the challenge experiment. The challenge dosage of VP_AHPND_ was 5 × 10^6^ CFU/mL, which was the half of half-lethal dose determined by pre-experiment. The *VP*_AHPND_ were added to the aquarium at a final concentration of 5 × 10^6^ CFU/mL. The hepatopancreas was dissected from three shrimp and mixed as one biological sample and three biological replicates were prepared at 0, 6 and 12 h post infection (hpi). The samples were pre-treated in liquid nitrogen and then stored at −80 °C.

### 2.2. Molecular Detection of V. parahaemolyticus in the Hepatopancreas

To explore the dynamic changes in *V. parahaemolyticus* in shrimp, DNA from the hepatopancreas samples was extracted using the Plant Genome DNA Extraction Kit (TIANGEN, Beijing, China) according to the manufacturer’s protocols. The concentration and purity of DNA was measured by NanoDrop 2000 (Thermo Fisher Scientific, Waltham, MA, USA), and the DNA integrity was measured by 1% agarose gel electrophoresis. Then, the extracted DNA was screened for the presence of the *PirA^Vp^* sequence using the TaqMan-probe fluorescence real-time PCR. The copy number of *PirA^Vp^* was calculated according to the standard curves, which were constructed by measuring the presence of the *PirA^Vp^* and *PirB^Vp^*. The primers used in RT-qPCR are shown in Table S1.

### 2.3. Total RNA Extraction, Library Preparation, and Transcriptome Sequencing

Total RNA from the hepatopancreas samples was extracted with RNAiso Plus (Takara, Japan) according to the manufacturer’s instructions. The quality and concentration of RNA samples were determined by 1% agarose gel electrophoresis and NanoDrop 2000 spectrophotometer (Thermo Fisher Scientific, Waltham, MA, USA). The mRNA was enriched by the magnetic beads with Oligo (dT) (New England Biolabs, MA, USA) and then fragmented into randomly short fragments with fragmentation buffer. Subsequently, the short mRNA fragments were used as templates for reverse transcription into the first-strand cDNA with random primers, and the second-strand cDNA was synthesized by adding DNA polymerase I, RNaseH, dNTP and buffer (New England Biolabs, USA). Then, the cDNA fragments were purified, end repaired, ploy(A) added and linked to Illumina sequencing adapters. The appropriate fragments were selected by agarose gel electrophoresis. Finally, PCR was used to complete the preparation of the library, and the constructed library was sequenced using Illumina HiSeqTM 2500 by Genedenovo Biotechnology Co., Ltd. (Guangzhou, China).

### 2.4. Transcriptome Assembly and Gene Functional Annotation

Before assembly, raw reads were filtered to remove reads containing adapters or more than 10% of unknown nucleotides (N), low-quality reads and rRNA, in order to obtain high-quality clean reads. Then, the clean reads were mapped to the reference genome [23] by HISAT2.2.4 [24]. The mapped reads were assembled using StringTie v 1.3.1 [25,26] in a reference-based approach. The reconstructed transcripts were aligned to the reference genome, and the functional annotation of novel genes was carried out by aligning to Nucleotide Sequence (NT), Non-Redundant protein sequence (NR), Swiss-Prot, Clusters of orthologous groups of proteins (COG) and Kyoto Encyclopedia of Genes and Genomes (KEGG) databases. To characterize the AHPND related genes in *Litopenaeus vannamei,* nine cDNA libraries representing samples at post-AHPND infection stages (6 hpi and 12 hpi) and non-AHPND-infected stage (0 hpi) were constructed. The sample 6 hpi-H-1 had a very low correlation with the other two samples in the 6 hpi group, which was even lower than the correlation between 6 hpi-H-1 and samples from other groups. There should be a contamination of other tissues in the sample 6 hpi-H-1 during tissue collection. Therefore, the sample 6 hpi-H-1 was removed, and eight samples were used for transcriptome analysis.

### 2.5. Identification of Differentially Expressed Genes (DEGs)

To identify the DEGs of 6 hpi vs. 0 hpi, 12 hpi vs. 0 hpi and 12 hpi vs. 6 hpi, the expression level of each transcript was quantified by calculating its FPKM (fragment per kilobase of transcript per million mapped reads). DEGs before and after infection were analyzed using the edgeR package (http://www.rproject.org/, accessed on 28 September 2020), with the parameter of false discovery rate (FDR) below 0.05 and absolute fold change ≥ 2. In addition, the correlation analysis between samples was also carried out among samples to ensure the reliability of experimental data.

### 2.6. Enrichment Analysis of DEGs

The Gene Ontology (GO) functions and KEGG pathways enrichment analysis of DEGs were performed using the OmicShare tools (http://www.omicshare.com/tools) (accessed on 28 September 2020). The GESA software v4.10 (https://www.gsea-msigdb.org/gsea/downloads.jsp) (accessed on 28 September 2020) [27] was used for Gene Set Enrichment Analysis (GESA) in two comparison groups. The *p*-value < 0.05 and Q ≤ 0.05 were considered statistically significant and were chosen for further analysis.

### 2.7. Validation of Differentially Expressed Genes by RT-qPCR

To validate the transcriptome data, twelve DEGs were selected randomly to perform a relative quantitative real-time PCR (RT-qPCR) analysis. The technical validation was conducted in the same RNA prepared for RNA-seq. The first strand of cDNA was reverse transcribed from RNA (1 μg) using PrimeScript™ RT Reagent Kit with gDNA Eraser (TaKaRa, Japan), and diluted by 30-fold in nuclease-free water. Primers for qRT-PCR were designed using Primer Premier 5.0 software. The primers used in RT-qPCR are shown in Table S1. 18s rRNA was used as an internal control to standardize the expression level. The RT-qPCR was conducted with THUNDERBIRD^®^ SYBR^®^ qPCR Mix on the Eppendorf Mastercycler ep realplex (Eppendorf, Germany) in a total volume of 10 μL, containing 5 μL of qPCR Mix, 1 μL of diluted cDNA, 0.3 μL each of forward and reverse primer, and 3.4 μL of DEPC-H_2_0. The amplification steps were as follows: 95 °C for 2 min; 40 cycles of 95 °C for 15 s, 56 °C for 15 s and 72 °C for 30 s; and followed by a melting curve. The RT-qPCR analysis of all templates was repeated three times. The RT-qPCR data of the transcription of DEGs were calculated by 2^−ΔΔct^.

## 3. Results

### 3.1. The Loads of V. parahaemolyticus in Shrimp Hepatopancreas

The copy number of *PirA^Vp^* per ng hepatopancreas DNA of shrimp at different infection time points was shown in Figure S1. The copy number of *PirA^Vp^* per ng hepatopancreas DNA was 0.25 cfu/ng at 0 hpi, 1.43 cfu/ng at 6hpi, and 5.81 cfu/ng at 12 hpi. The copy number of *PirA^Vp^* did not change apparently during the early infection stages (0 hpi and 6 hpi), but was significantly increased from 6 hpi to 12 hpi, indicating that the *V. parahaemolyticus* proliferated significantly in hepatopancreas from 6 hpi to 12 hpi.

### 3.2. Transcriptome Sequencing Data

The details of hepatopancreas transcriptome sequencing and assembly of *Litopenaeus vannamei* are presented in Table S2. A total of 454,180,184 raw reads were obtained. After filtering out low-quality data, a total of 452,336,166 clean reads were obtained. The proportion of each sample with quality scores ≥ Q20 and ≥ Q30 exceeded 97% and 92%. After mapping to the reference genome, a total of 25,572 unigenes were obtained, of which 7912 were newly assembled.

### 3.3. Differentially Expressed Genes (DEGs) in Different Comparisons

**Figure 1:**
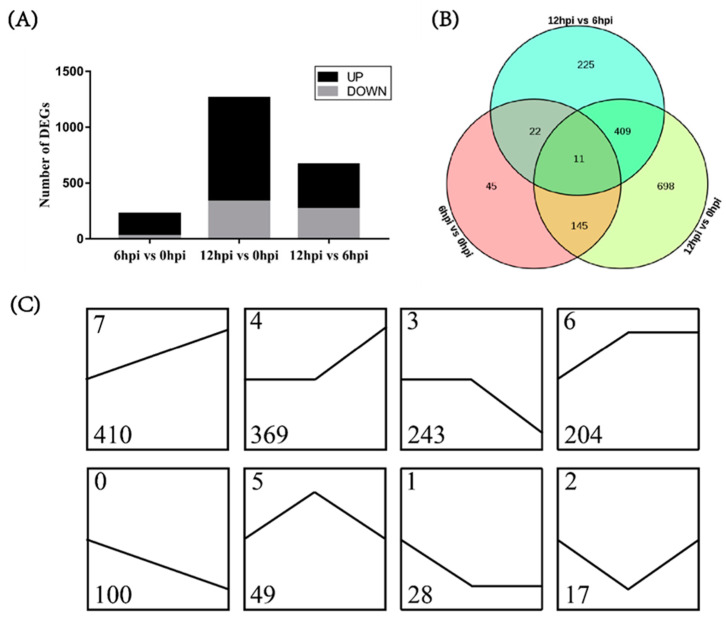
Identification of differentially expressed genes (DEGs) from the transcriptome data. (**A**) Comparison of DEGs between three pairwise groups. Up represents upregulated genes. Down represents downregulated genes. (**B**) Venn diagrams for three pairwise comparisons at different time points after *V. parahaemolyticus* infection. (**C**) Trend analysis of DEGs. The number of genes with the same trend was marked in the lower left corner of each profile. Profiles were ordered based on the number of genes assigned.

The expression levels of unigenes between two different time points were compared by FPKM value to identify DEGs. A total of 223 DEGs were obtained in the 6 hpi vs. 0 hpi group, including 199 upregulated unigenes and 24 downregulated unigenes (Figure 1A). A total of 1263 DEGs were obtained in the 12 hpi vs. 0 hpi group, including 931 upregulated unigenes and 332 downregulated unigenes. There were 156 DEGs that were significantly different between the two comparison groups. A total of 667 DEGs were obtained in the 12 hpi vs. 6 hpi group, including 399 upregulated unigenes and 268 downregulated unigenes.

To further understand the relationship between DEGs in pairwise comparison groups, we identified the common DEGs. As shown in the Venn diagram (Figure 1B), few genes were shared among the three comparison groups, while 420 common DEGs were shared by the 12 hpi vs. 0 hpi group and the 12 hpi vs. 6 hpi group. This phenomenon might be related to the proliferation process of *V. parahaemolyticus* in the hepatopancreas. Furthermore, we analyzed the expression patterns of DEGs and grouped them into eight categories based on the trend of gene expression (Figure 1C). By counting the gene number of each category, we found that most of the DEGs were upregulated after *V. parahaemolyticus* infection (profile 7, profile 4, profile 6 and profile 5). These DEGs were mainly related to metabolism, signal pathway, cancers and diseases (Table S3). Interestingly, many of the upregulated genes were key regulatory genes in the metabolic pathways, such as glucose metabolism, amino acid metabolism, and nucleotide metabolism. Moreover, the upregulated expression of genes in metabolic pathways mainly occurred from 6 hpi to 12 hpi, when *V. parahaemolyticus* proliferated significantly in the hepatopancreas. Therefore, it was considered that the alterations in host metabolism were responsible for the massive proliferation of *V. parahaemolyticus.*

### 3.4. Functional Annotation of DEGs

**Figure 2:**
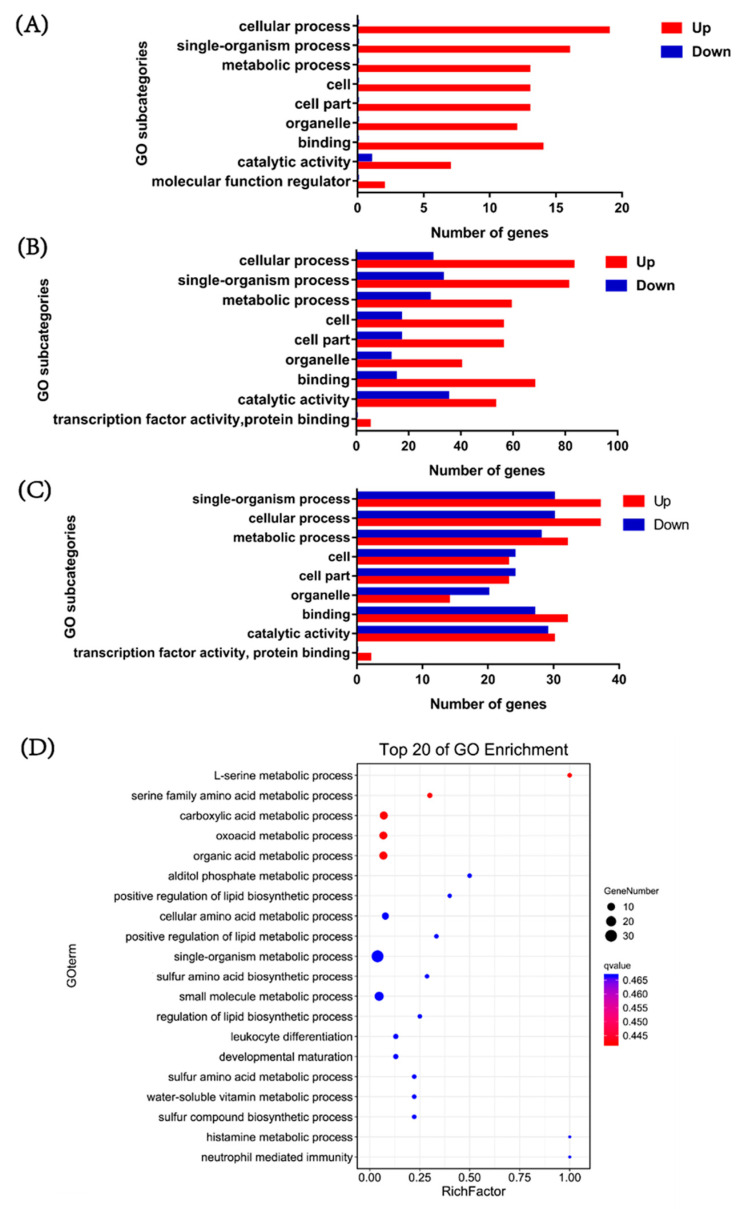
Gene ontology (GO) functional enrichment analysis of DEGs in pairwise comparisons between three time points. The column showed quantity of up(red)- and down(blue)-regulated DEGs. (**A**) Enriched GO items from the 6 hpi vs. 0 hpi comparison. (**B**) Enriched GO items from the 12 hpi vs. 0 hpi comparison. (**C**) Enriched GO items from the 12 hpi vs. 6 hpi comparison. (**D**) The most enriched biological processes from the 12 hpi vs. 6 hpi comparison.

To investigate the function of DEGs in response to *V. parahaemolyticus* infection, gene ontology (GO) functional enrichment analysis was performed in three comparison groups. In the biological process category, DEGs were mainly enriched in the cellular process, signal-organism and metabolic process (Figure 2A). In the cellular component, most DEGs were enriched in the cell, cell part and organelle (Figure 2B). In the molecular function category, most DEGs were involved in binding and catalytic activity (Figure 2C). According to the number of DEGs in different subcategories, the changes in the processes involved in these major subcategories mainly occurred at 6–12 h after *V. parahaemolyticus* infection. In particular, the most enriched process was the metabolic process in the biological process, including L-serine metabolic process, serine family amino acid metabolic process, carboxylic acid metabolic process, oxoacid metabolic process, organic acid metabolic process, alditol phosphate metabolic process, positive regulation of lipid biosynthetic process, cellular amino acid metabolic process and positive regulation of lipid metabolic process (Figure 2D). The results indicated that metabolic processes might play important roles during *V. parahaemolyticus* infection.

**Figure 3:**
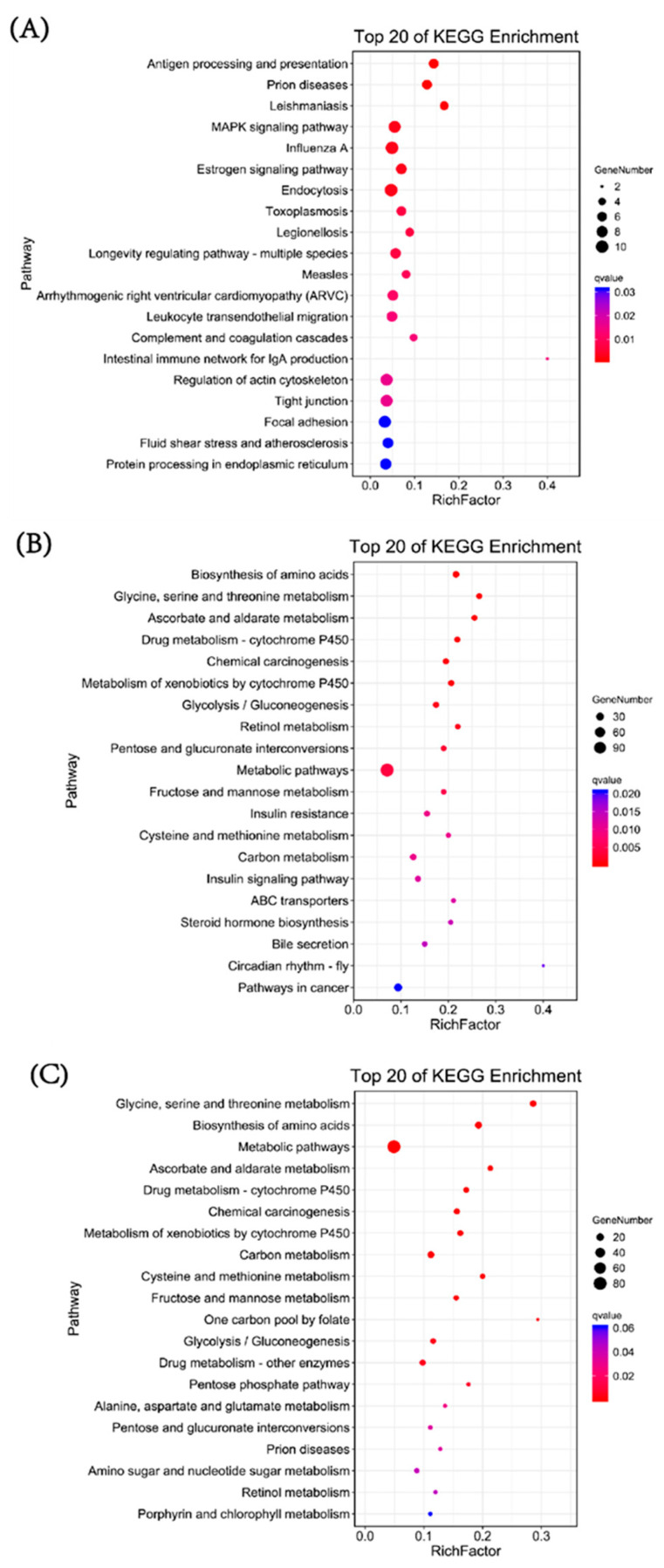
KEGG pathway enrichment analysis of DEGs in pairwise comparisons between three time points. (**A**) Enriched KEGG pathways from the 6 hpi vs. 0 hpi comparison. (**B**) Enriched KEGG pathways from the 12 hpi vs. 0 hpi comparison. (**C**) Enriched KEGG pathways from the 12 hpi vs. 6 hpi comparison.

KEGG pathway enrichment analysis was conducted to investigate the specific pathways involved in DEGs. In the 6 hpi vs. 0 hpi group, the most enriched pathways of DEGs were antigen processing and presentation, prion diseases, leishmaniasis, MAPK signaling pathway, influenza A, estrogen signaling pathway, endocytosis, toxoplasmosis, legionellosis and longevity regulating pathway-multiple species (Figure 3A). In the 12 hpi vs. 0 hpi group and 12 hpi vs. 6 hpi group, there were many enrichment pathways that were the same, including biosynthesis of amino acids, glycine, serine and threonine metabolism, ascorbate and aldarate metabolism, drug metabolism-cytochrome P450, chemical carcinogenesis, metabolism of xenobiotics by cytochrome P450, glycolysis/gluconeogenesis, retinol metabolism, pentose and glucuronate interconversions, metabolic pathways, fructose and mannose metabolism, cysteine and methionine metabolism, carbon metabolism and alanine, aspartate and glutamate metabolism (Figure 3B,C). These pathways are basically related to the metabolism of substances (mainly glucose and amino acids), and some were related to detoxification. Significant changes in the expression of genes involved in these metabolic pathways mainly occurred during 6 hpi to 12 hpi, when *V. parahaemolyticus* proliferated rapidly in the host. These results suggest that the changes in host metabolism are mainly caused by the massive proliferation of *V. parahaemolyticus.*

### 3.5. Screening and Trend Analysis of Metabolism-Related DEGs

**Figure 4:**
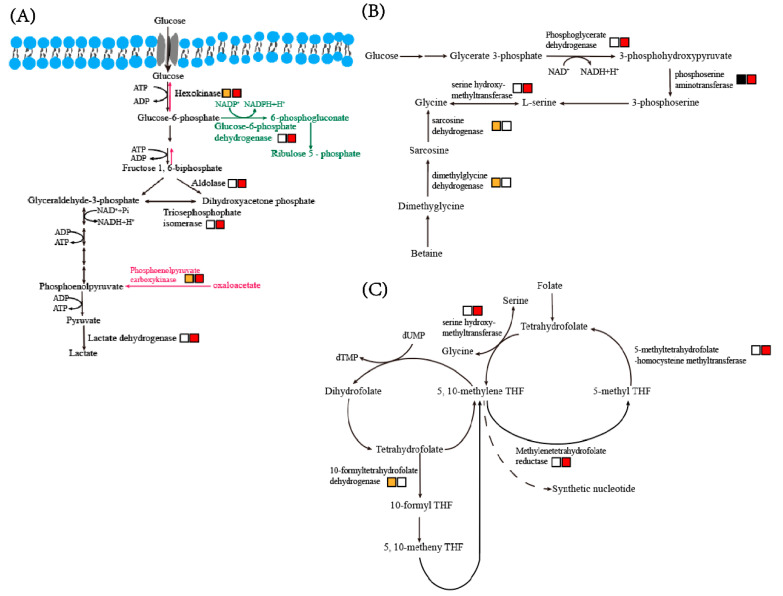
Expression trends of DEGs involved in metabolic pathways. (**A**) Glycolysis, gluconeogenesis (pink) and pentose phosphate pathway (green). (**B**) Glycine, serine and threonine metabolism. (**C**) One carbon pool by folate. The box shows the upregulated DEGs from the 6 hpi vs. 0 hpi comparison (orange), the upregulated DEGs from the 12 hpi vs. 0 hpi comparison (red) and the downregulated DEGs from the 6 hpi or 12 hpi vs. 0 hpi comparisons (black).

To understand the metabolic changes in the hepatopancreas of shrimp with AHPND, we screened genes involved in metabolic processes and analyzed their expression trends. Most of the DEGs were found to encode the key enzymes of key metabolic pathways, including glycolysis, gluconeogenesis pathway, pentose phosphate pathway, glycine, serine and threonine metabolism, and one carbon pool by folate (Figure 4 and Figure S2).

#### 3.5.1. Glycolysis

*V. parahaemolyticus* infection led to a significant increase in the expression of genes involved in the glycolysis pathway (Figure 4A). Hexokinase (HK), the first rate-limiting enzyme of glycolysis, was significantly upregulated at 6 hpi and 12 hpi. The expression level of fructose 1,6-biphosphate-aldolase (FBA) gradually increased 8-fold from 0 hpi to 12 hpi. Several homologous genes encoding triosephosphate isomerase were significantly upregulated at 12 hpi. Lastly, the transcriptional level of lactate dehydrogenase (LDH) increased significantly at 12 hpi.

#### 3.5.2. Gluconeogenesis and Pentose Phosphate Pathway

The expression level of phosphoenolpyruvate carboxykinase (PCK), the rate-limiting enzyme of the gluconeogenesis pathway, gradually increased after VP_AHPND_ infection (Figure 4A). Glucose-6-phosphate dehydrogenase (G6PDH), the rate-limiting enzyme of the pentose phosphate pathway, showed significant upregulation at 12 hpi (Figure 4A). The upregulation of PCK indicated that large amounts of intermediate metabolites would be generated with the progress of infection, providing substrates for amino acid synthesis. The products catalyzed by G6PDH would also be used as substrates for de novo synthesis of nucleic acids.

#### 3.5.3. Glycine, Serine and Threonine Metabolism

PHGDH encodes 3-phosphoglycerate dehydrogenase, the first branch enzyme of the glycolysis-serine biosynthetic pathway, was significantly upregulated at 12 hpi (Figure 4B). In addition, other enzymes involved in this pathway, including phosphoserine aminotransferase, serine hydroxymethyltransferase (SHMT), sarcosine dehydrogenase and dimethylglycine dehydrogenase, were also transcriptionally upregulated (Figure 4B). The expression levels of phosphoserine aminotransferase and serine hydroxymethyltransferase were significantly increased from 6 hpi to 12 hpi. The sarcosine dehydrogenase and dimethylglycine dehydrogenase were upregulated at 6 hpi.

#### 3.5.4. One Carbon Pool by Folate

There are four DEGs involved in the one carbon pool by folate pathway, including serine hydroxymethyltransferase, methylenetetrahydrofolate reductase (MTHFR), 5-methyltetrahydrofolate-homocysteine methyltransferase and formyltetrahydrofolate dehydrogenase (Figure 4C). The expression levels of formyltetra-hydrofolate dehydrogenase increased significantly at 6 hpi. The expression levels of the other three enzymes were significantly upregulated at 12 hpi. 

### 3.6. Enriched Signaling Pathways

Genes involved in HIF-1 signaling pathway, PI3K-Akt signaling pathway and NF-kappa B signaling pathway were also upregulated in hepatopancreas after AHPND infection.

#### 3.6.1. HIF-1 Signaling Pathway

**Figure 5:**
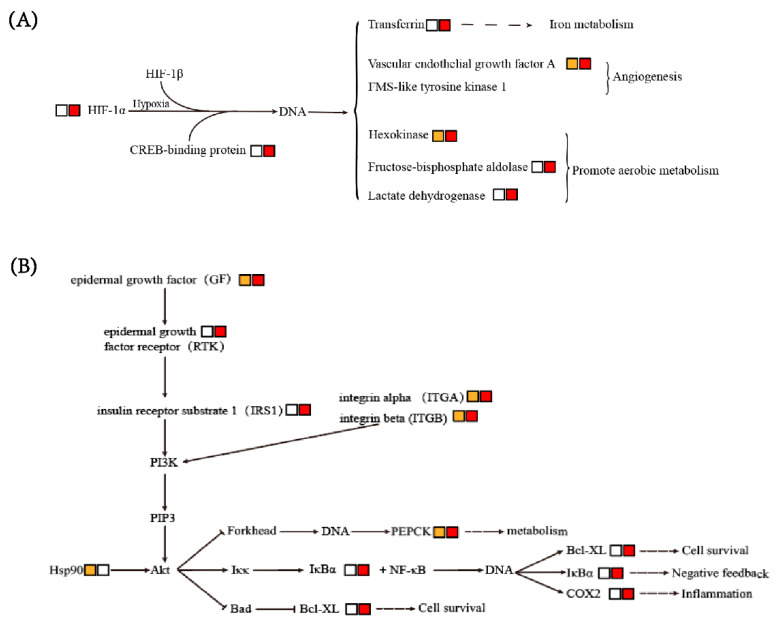
Expression trends of DEGs involved in PI3K-Akt (**A**) and NF-κB (**B**) signaling pathways. The box showed upregulated DEGs from the 6 hpi vs. 0 hpi comparison (orange), the upregulated DEGs from the 12 hpi vs. 0 hpi comparison (red) and the downregulated DEGs from the 6 hpi or 12 hpi vs. 0 hpi comparisons (black).

In the HIF-1 signaling pathway, HIF-1α, the active subunit of the transcriptional activator HIF-1 [28], was significantly upregulated after *V. parahaemolyticus* infection (Figure 5A). The HIF-1 activated genes involved in iron metabolism, angiogenesis and anaerobic metabolism, including transferrin (TF), vascular endothelial growth factor A (VEGFA), vascular endothelial growth factor receptor precursor/FMS-like tyrosine kinase 1 (VEGFR1 or FLT1), hexokinase (HK), fructose 1,6-biphosphate-aldolase A (ALDOA), and lactate dehydrogenase A (LDHA), were also upregulated after *V. parahaemolyticus* infection (Figure 5A).

#### 3.6.2. PI3K-Akt and NF-κB Signaling Pathways

In PI3K-Akt and NF-κB signaling pathways, genes encoding epidermal growth factor (GF), epidermal growth factor receptor (RTK), insulin receptor substrate 1 (IRS1), integrin, heat shock protein 90 (HSP90), Bcl-XL, phosphoenolpyruvate carboxykinase (PCK), cyclooxygenase (COX2), and cactus protein (IκBα) all showed significant upregulation after *V. parahaemolyticus* infection (Figure 5B). The above-mentioned genes are involved in different cell activities, such as cell survival, anaerobic metabolism and inflammation.

### 3.7. Verification of RNA-seq Results by RT-qPCR

**Figure 6:**
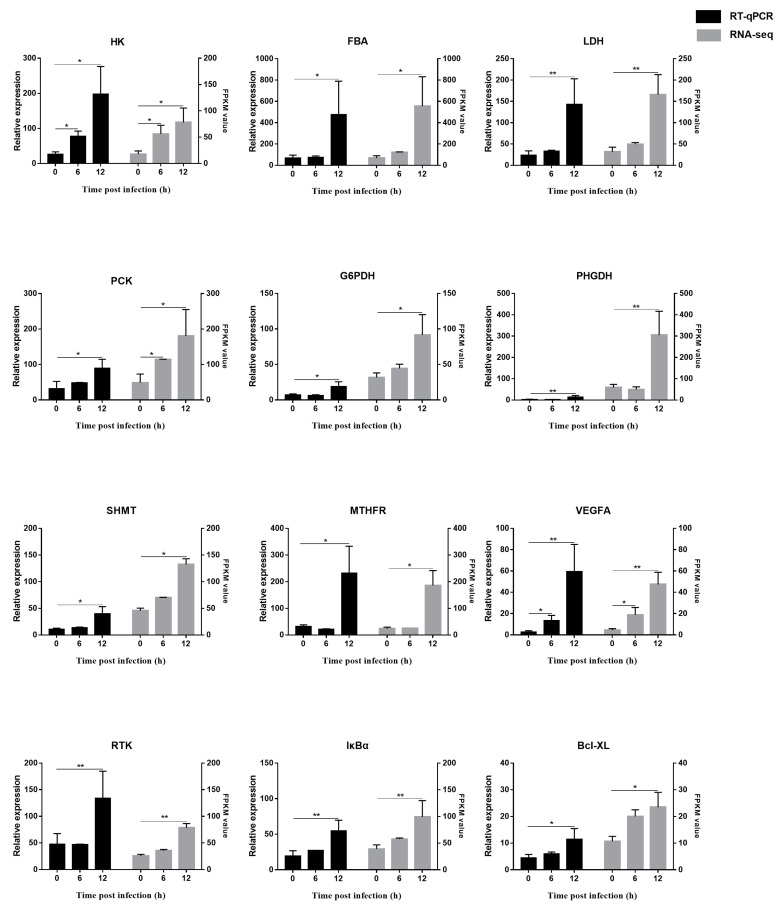
RT-qPCR and RNA-seq results of twelve DEGs in hepatopancreas. The data were calculated as the mean ± SD relative to the reference gene (18S rRNA). The significant statistical difference was marked with * (*p* < 0.05) or ** (*p* < 0.01).

A total of twelve DEGs in which transcriptional levels changed significantly after *V. parahaemolyticus* infection were chosen for RT-qPCR to validate the RNA-seq results. For the twelve DEGs acquired from three comparison groups, they were all verified successfully in the hepatopancreas. As shown in Figure 6 and Figure S2, the expression trends of these DEGs which participated in metabolic and signaling pathways were exactly consistent with the transcriptome data.

## 4. Discussion

Recently, studies have shown that the interaction between immunity and metabolism seems to participate in shrimp’s response to AHPND [29]. However, transcriptome studies of shrimp responses to AHPND have mainly focused on immune-related genes and pathways, and little is known about metabolism-related changes [16,30,31,32]. Here, the comparative transcriptome study mainly focused on the metabolic processes in shrimp hepatopancreas at different time points post *V. parahaemolyticus* infection. The data revealed that the expression levels of several genes that are involved in glucose metabolism, amino acid metabolism and nucleic acid metabolism were significantly affected after *V. parahaemolyticus* infection. In addition, the expression levels of some genes in the HIF-1 signaling pathway, PI3K-Akt signaling pathway and NF-kappa B signaling pathway, which participate in the regulation of metabolic processes, were also changed after *V. parahaemolyticus* infection.

The glycolysis/gluconeogenesis pathway is central to the metabolism of organisms, and its vital function is to provide energy and biosynthetic precursors such as amino acids, lipids and nucleotides [33]. Under aerobic conditions, glycolysis is a catabolic process that catalyzes the conversion of glucose to pyruvate via ten enzymatic steps [34]. Subsequently, the pyruvate is metabolized through the TCA cycle into NADH and reduced FADH_2_ to perform oxidative phosphorylation. However, pyruvate can also be fermented to lactate without producing ATP but regenerating NAD^+^ [35]. This phenomenon was known as Warburg effect in mammalian [36]. The excessive consumption of glucose and accumulation of lactate are both hallmarks of the Warburg effect [37]. The Warburg effect has been widely accepted as a common feature of metabolic reprogramming. Enhanced glycolysis, blocked tricarboxylic acid (TCA) cycle and oxidative phosphorylation (OXPHOS) are all key features of metabolic reprogramming in cancer cells [34]. In some studies, researchers also found that pathogens could cause these changes by exploiting the metabolic pathways of hosts to meet their raw material needs [38,39,40]. In the present study, we found that the hexokinase (HK) and lactate dehydrogenase (LDH), two key enzymes of metabolic reprogramming [41,42], were both significantly upregulated after *V. parahaemolyticus* infection. The expression of HK is often upregulated in cancer cells, and it plays a vital role in the high glycolytic phenotype [43,44]. LDH is involved in both aerobic and anaerobic glycolysis, which could also be induced by the hypoxia inducible factor-1 (HIF-1) [45,46]. The high expression of these two enzymes and other enzymes in the glycolysis pathway suggested that *V. parahaemolyticus* infection significantly enhanced the glycolysis of shrimp, causing rapid production of ATP. Notably, the expression levels of LDH did not increase significantly at 6 hpi, but increased significantly at 12 hpi, indicating that *V. parahaemolyticus* infection might cause significant changes in glycolysis during the rapid proliferation of *Vibrio*. This phenomenon may be related to the fact that *V. parahaemolyticus* initially colonizes the stomach at the early infection stage, and then released the PirA- and PirB-like toxins to the hepatopancreas [47,48]. The energy produced might not only be used to fight infection and maintain homeostasis by the host, but might also be used for *Vibrio* proliferation, similar to how viruses use glycolysis for successful replication in shrimp [39]. In addition, with the consumption of glucose, the gluconeogenesis pathway was also enhanced, with the most obvious feature that the upregulation of PCK at 6 hpi and 12 hpi, a key regulatory enzyme of gluconeogenesis. Additionally, the glycolysis pathway also provides precursors for substance synthesis, such as nucleic acids, amino acids and fatty acids [49]. Therefore, the activation of glycolysis might also enhance other biosynthetic processes.

The pentose phosphate pathway (PPP) branches from the first step of glycolysis. This pathway has two important functions, providing precursors for nucleotide synthesis (which often occurs during cell rapid proliferation) and balancing cellular redox conditions [50]. After *V. parahaemolyticus* infection, PPP was significantly enhanced with the phenomenon of G6PDH upregulation. G6PDH is the first and rate-limiting enzyme and as a control enzyme in the oxidative branch of the PPP, responsible for the ribose-5-phosphate and NADPH generation. NADPH is involved in many processes, including detoxification of intracellular reactive oxygen species (ROS), reductive biosynthesis and ribose biogenesis [51]. In crustaceans, ROS are important factors in the immunity of shrimp against microbes and virus infection [52,53,54]. The upregulation of PPP from 6 hpi to 12 hpi was consistent with the proliferation of *Vibrio* in shrimp. Therefore, the changes in PPP after infection might neutralize the ROS produced by the shrimp and facilitate the replication of *Vibrio*.

Glycine, serine and threonine metabolism is the most significantly enriched amino acid metabolic pathway after *Vibrio* infection. In this pathway, the enzymes involved in de novo synthesis of serine (SSP) and serine conversion to glycine were significantly increased from 6 hpi to 12 hpi after infection. Serine is a non-essential amino acid, derived from the glycolytic intermediate, 3- phosphoglycerate (3-PG), and can be converted to glycine [55]. Serine and glycine are both sources of one-carbon units. Serine can be converted to glycine by SHMT1/2 and one-carbon units for the synthesis of nucleotides, proteins, lipids and metabolites. Glycine can produce one-carbon units via a glycine cleavage system [56]. One-carbon units are essential for biosynthetic pathways, methylation modification and synthesis of metabolites, such as NAD(P)H, ATP and SAM/SAH [55]. The two pathways involved in the metabolism of one carbon unit are folate acid cycle and methionine metabolism [57]. In the present study, the expression levels of dimethylglycine dehydrogenase, sarcosine dehydrogenase, methylenetetrahydrofolate reductase and 10-formyltetrahydrofolate dehydrogenase, which are responsible for the folate cycle, increased significantly after *Vibrio* infection. The results indicated that *Vibrio* infection might cause disorders of the synthesis of nucleotides, proteins, lipids and metabolites, and affect the survival and proliferation of hepatopancreas cells. In *L. vannamei*, aflatoxins produced by fungi cause changes in serine metabolism, which was used to resist the damaging effects of the toxin [58]. However, serine/glycine biosynthesis and one-carbon metabolism also contribute to the survival and rapid proliferation of cancer cells [59]. Therefore, it is still ambiguous and needs to clarify whether the disorder of this pathway facilitates hepatopancreas cells to clear *Vibrio* toxins or benefits *Vibrio* to promote their own proliferation.

The responses of HIF-1, PI3K-Akt and NF-kappa B signaling pathways to *Vibrio* infection further supported the transcriptional changes in these metabolic processes. During hypoxia conditions, animals usually utilize the anaerobic glycolysis to satisfy their energy requirement. HIF-1 is one of the main responsible factors for coordinating anaerobic metabolism [60]. The target genes of HIF-1 are involved in a variety of cellular processes, such as glycolysis, erythropoiesis, angiogenesis and vascular remodeling [61]. In our transcriptome data, the genes of the HIF-1 signaling pathway were significantly upregulated after *Vibrio* infection, indicating that the infection and proliferation of *Vibrio* might alter the microenvironment of hepatopancreas cells, resulting in oxygen deficiency and thereby inducing HIF-1 signaling pathway. According to many studies, the hypoxic induction of HIF-1α protein could be regulated by PI3K/Akt signaling pathway [62]. PI3K/Akt signaling pathway plays an important role in various normal activities of cells, including cell proliferation, differentiation, survival, metabolism and motility [63]. In the present study, the genes involved in the PI3K/Akt signaling pathway and the downstream target genes of the NF-κB signaling pathway were also significantly upregulated after *Vibrio* infection. Overall, *Vibrio* infection might cause oxygen deficiency, leading to the upregulation of these signaling pathways and the enhancement of anaerobic glycolysis in hepatopancreatic cells. These changes play an important role in cell survival and resistance to *Vibrio* infection.

In summary, we compared the gene expression profiles of three time points of *L. vannamei* before and after VP_AHPND_ infection. The DEGs were significantly enriched in the metabolism pathway and related signaling pathways, including the glycolysis/gluconeogenesis pathway, the pentose phosphate pathway, serine/glycine metabolism, one-carbon unit metabolism, HIF-1 signaling pathway, PI3K-Akt signaling pathway and NF-kappa B signaling pathway. Compared with the uninfected shrimp, these metabolic pathways were disordered after infection, similar to the metabolic reprogramming in cancer cells and immune cell functional changes during the pro- and anti-inflammatory phases [64]. Changes in these pathways not only contribute to host resistance to *Vibrio* infection, but can also be plundered by *Vibrio* for its own proliferation. The results provide us with new insights on finding anti-*Vibrio* strategies based on these metabolic pathways.

## 5. Conclusions

Comparative transcriptome analysis on hepatopancreas of *Litopenaeus vannamei* indicated that the metabolic processes and related signaling pathways of shrimp changed significantly at different time points after *Vibrio parahaemolyticus* infection. The DEGs of metabolic processes mainly consisted of glucose, energy and amino acid metabolism, as well as nucleic acid synthesis. The signaling pathways, including HIF-1 signaling pathway, PI3K-Akt signaling pathway and NF-KappaB signaling pathway, which participate in the modulation of metabolic processes, were also responsive to *V. parahaemolyticus* infection. Moreover, the significant changes in host metabolism were consistent the proliferation of *VP*_AHPND_, indicating that the metabolism dysregulation might be related to *Vibrio* proliferation. The results revealed a close relationship between host metabolism and *Vibrio* proliferation, which might help to develop anti-*vibrio* strategies from the metabolic perspective.

## Data Availability

The original contributions presented in the study are included in the article/Supplementary Material. Further inquiries can be directed to the corresponding authors.

## Supplementary Materials

The following supporting information can be downloaded at: https://www.mdpi.com/article/10.3390/biology12030417/s1, Figure S1: Copy number of *PirA^Vp^* per ng hepatopancreas DNA at 0 hpi, 6 hpi and 12 hpi. The statistic significant difference was indicated with * (*p* < 0.05), Figure S2: Heatmap visualization of the expression trends of DEGs involved in metabolic processes and signaling pathways among different time points, Table S1: Primers and probes used in the present study, Table S2: Summary of the transcriptome data, Table S3: KEGG enrichment analysis of profile 7, 4, 6 and 5, Table S4: Pearson correlation coefficient analysis between the results of RNA seq and RT-qPCR for twelve DEGs.
