# Supplementary material for: Transcriptome Analysis on Hepatopancreas Reveals the Metabolic Dysregulation Caused by Vibrio parahaemolyticus Infection in Litopenaeus vannamei
Source: Biology (Basel). 2023 Mar 9;12(3):417. doi: 10.3390/biology12030417 (PMC10044748; doi:10.3390/biology12030417)
Supplement: Supplementary file 1 [file biology-12-00417-s001.zip › Figure S1.pdf]

Figure S1 Copy number of *$PirA^{Vp}$* per ng hepatopancreas DNA at 0hpi, 6hpi and 12hpi. The statistic significant difference is indicated with "*" ($p < 0.05$).

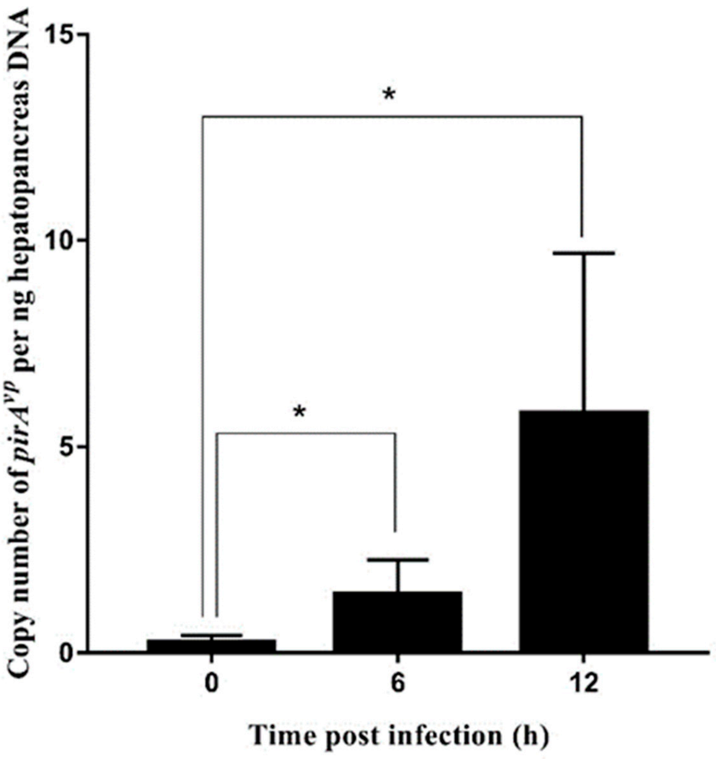

Figure S1
