# Supplementary material for: Transcriptome Analysis on Hepatopancreas Reveals the Metabolic Dysregulation Caused by Vibrio parahaemolyticus Infection in Litopenaeus vannamei
Source: Biology (Basel). 2023 Mar 9;12(3):417. doi: 10.3390/biology12030417 (PMC10044748; doi:10.3390/biology12030417)
Supplement: Supplementary file 1 [file biology-12-00417-s001.zip › Figure S2.pdf]

**Figure S2** Heatmap visualization of the expression trends of DEGs involved in metabolic processes and signaling pathways from 0hpi to 6hpip and 12hpi.

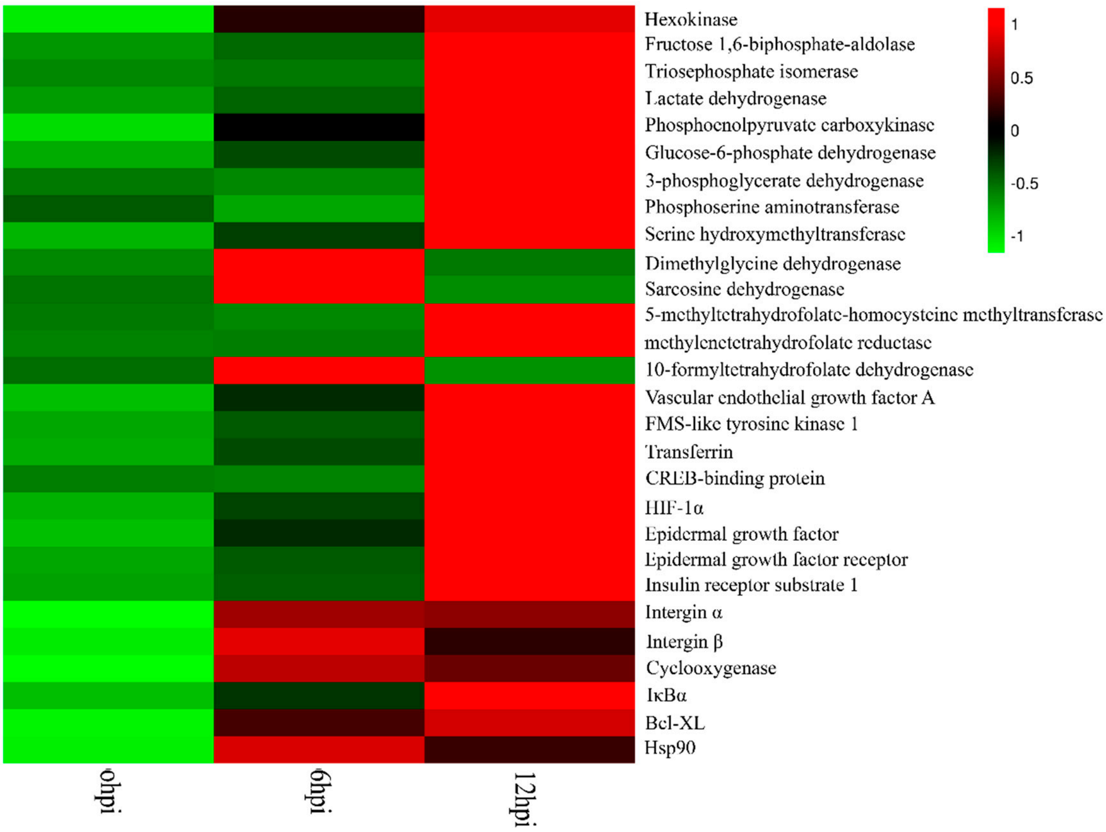


**Figure S2**
