# Supplementary material for: Transcriptome Analysis on Hepatopancreas Reveals the Metabolic Dysregulation Caused by Vibrio parahaemolyticus Infection in Litopenaeus vannamei
Source: Biology (Basel). 2023 Mar 9;12(3):417. doi: 10.3390/biology12030417 (PMC10044748; doi:10.3390/biology12030417)
Supplement: Supplementary file 1 [file biology-12-00417-s001.zip › Table S1 Primers and probes used in this paper.pdf]

Table S1 Primers and probes used in this paper

Primers and probes used for TaqMan-Probe fluorescence quantitative PCR			
**Gene**	**Primer sequence (5'-3')**	**Probe sequence (5'-3')**	**Modification of probe**
PirA^{Vp}	F:CGGAAGTCGGTCGTAGTGTA<br>R:TGTGATTTAGCCACTTTCCAG	CCGCCAGCCATAAATGGCGC ACC	5'6-FAM<br>3'BHQI
PirB^{Vp}	F: TGAAGTGATGGGTGCTCGTA<br>R:ACCAACAGCAGGTGAATATGA	TGTTCAAAGCCGTGAACCGT ACACCA	5'HEX<br>3'BHQ1

The primers used for validation of DEGs by RT-qPCR

**Gene**	**Primer sequence (5'-3')**	**Expected size (bp)**	**Annealing temperature (℃)**
**HK**	F:TGAGTGTCTCGCAAGCTTCA<br>R: TTGTGCCAGTGCCAACAATC	**311**	**57**
**FBA**	F: AGCCGACGAGTCTGTCTCTA<br>R: TTCGTGGAAGAGGATCACGC	**151**	**57**
**LDH**	F: TTGTTACTGCTGGTGCTCGT<br>R: AGCGACACTCAGTTTCTGGG	**266**	**57**
**PCK**	F: ACATCTGCGATGGAAGCGAA<br>R: CGGTCCTTGGTCACGATGAA	**166**	**57**
**G6PDH**	F: ACTTCGAGCTGCTGGACCAG<br>R: CGAACGGCTTCTCGATGATC	**167**	**57**
**PHGDH**	F: ACTCTACGGGAAGACCCTCG<br>R: CTTGCTCAAGACCTCCGACC	**247**	**57**
**SHMT**	F:TCGGAGAACTTCGCATCTCG<br>R: GTTGCACATTGACACCCCAC	**193**	**57**
**MTHFR**	F: AAGCAGTGAGGACCCAGTTG<br>R: CATTGGACGGTGTTGATGGC	**199**	**57**
**VEGFA**	F: ACAAGATGGTCGAGAACCCG<br>R: CGTAACGACATTCGCACGTT	**129**	**57**
**RTK**	F: TTCATGATGGGCAAACCGGA<br>R: TGATTGGCAACGGTAGGGAC	**227**	**57**
**IκBα**	F: CGGGTTTCCTGTCTGGTTCA<br>R: TCGTGCTCGAGGATGAGGTA	**228**	**57**
**Bcl-XL**	F: ATATCACACGCGAGACAGGC<br>R: TGGCCTCCAAAGGCATAGAG	**118**	**57**
