# Supplementary material for: Transcriptome Analysis on Hepatopancreas Reveals the Metabolic Dysregulation Caused by Vibrio parahaemolyticus Infection in Litopenaeus vannamei
Source: Biology (Basel). 2023 Mar 9;12(3):417. doi: 10.3390/biology12030417 (PMC10044748; doi:10.3390/biology12030417)
Supplement: Supplementary file 1 [file biology-12-00417-s001.zip › Table S2 Summary of transcriptome sequencing data.pdf]

Table S2 Summary of transcriptome sequencing data

Groups	Sample	Raw reads	Clean reads	Raw bases	Clean bases	Q20(%)	Q30(%)
0h	0hpi-H-1	56183678	55981848	8427551700	8362771386	97.84%	93.96%
	0hpi-H-2	61716326	61457680	9257448900	9171277962	97.57%	93.32%
	0hpi-H-3	46799636	46652134	7019945400	6959313377	97.59%	93.29%
6hpi	6hpi-H-2	62128860	61881452	9319329000	9249288017	97.71%	93.56%
	6hpi-H-3	50434366	50307506	7565154900	7516556871	97.90%	94.00%
12hpi	12hpi-H-1	62171558	61851792	9325733700	9223310291	97.44%	93.11%
	12hpi-H-2	57670954	57388486	8650643100	8562124522	97.38%	92.92%
	12hpi-H-3	57074806	56815268	8561220900	8473326248	97.73%	93.76%
