# Supplementary material for: Transcriptome Analysis on Hepatopancreas Reveals the Metabolic Dysregulation Caused by Vibrio parahaemolyticus Infection in Litopenaeus vannamei
Source: Biology (Basel). 2023 Mar 9;12(3):417. doi: 10.3390/biology12030417 (PMC10044748; doi:10.3390/biology12030417)
Supplement: Supplementary file 1 [file biology-12-00417-s001.zip › Table S3 KEGG enrichment analysis of profile 7ú1⁄44ú1⁄4 6ú1⁄45.pdf]

Table S3 KEGG enrichment analysis of profile 7，4， 6，5

The significant enrichment pathways in profile 7		
**Pathway**	**Pvalue**	**Qvalue**
**Fructose and mannose metabolism**	0.000304221	0.03246635
**Insulin resistance**	0.00036804	0.03246635
**Glycolysis / Gluconeogenesis**	0.000425323	0.03246635
**Adipocytokine signaling pathway**	0.000573186	0.03281489
**Pathways in cancer**	0.001702217	0.07144434
**PI3K-Akt signaling pathway**	0.002020915	0.07144434
**HIF-1 signaling pathway**	0.002183888	0.07144434
**Circadian rhythm**	0.002750409	0.07732658
**ABC transporters**	0.003039036	0.07732658
**Proximal tubule bicarbonate reclamation**	0.006362653	0.1369491
**Toll and Imd signaling pathway**	0.006578341	0.1369491
**Rap1 signaling pathway**	0.00805033	0.14325379
**Human papillomavirus infection**	0.008132311	0.14325379
**Central carbon metabolism in cancer**	0.009394556	0.15366809
**Circadian rhythm - fly**	0.01055723	0.16117371
**Leishmaniasis**	0.01190712	0.17042066
**Biosynthesis of amino acids**	0.01322803	0.17818935
**Small cell lung cancer**	0.01623437	0.20653726
**MicroRNAs in cancer**	0.01902227	0.22926841
**Other glycan degradation**	0.02582822	0.27513478
**Ras signaling pathway**	0.02617626	0.27513478
**Carbon metabolism**	0.02697804	0.27513478
**Human cytomegalovirus infection**	0.02832354	0.27513478
**Pyruvate metabolism**	0.02933168	0.27513478
**Transcriptional misregulation in cancers**	0.03034831	0.27513478
**Focal adhesion**	0.03123801	0.27513478
**Cell adhesion molecules (CAMs)**	0.03307406	0.28051703
**Glucagon signaling pathway**	0.03470836	0.2838648
**Bile secretion**	0.03912906	0.30898465
**Autophagy - animal**	0.04586105	0.35007268

The significant enrichment pathways in profile 4		
**Pathway**	**Pvalue**	**Qvalue**
**Biosynthesis of amino acids**	0.000213135	0.03772491
**Carbon metabolism**	0.003767717	0.26463889
**Protein digestion and absorption**	0.004485405	0.26463889
**Nicotinate and nicotinamide metabolism**	0.009029971	0.34885319
**Sulfur metabolism**	0.00985461	0.34885319
**Notch signaling pathway**	0.01813458	0.42859665
**Insulin signaling pathway**	0.01875588	0.42859665
**Endometrial cancer**	0.0193716	0.42859665

**One carbon pool by folate**	0.02307632	0.45383429
**Arrhythmogenic right ventricular cardiomyopathy (ARVC)**	0.04166647	0.72867876
**Fructose and mannose metabolism**	0.04735545	0.72867876
**Cholesterol metabolism**	0.04940195	0.72867876
**The significant enrichment pathways in profile 6**		
**Pathway**	**Pvalue**	**Qvalue**
**Arrhythmogenic right ventricular cardiomyopathy (ARVC)**	2.60E-05	0.002718646
**Leukocyte transendothelial migration**	3.55E-05	0.002718646
**Regulation of actin cytoskeleton**	5.48E-05	0.002795713
**Platelet activation**	0.000305512	0.009424574
**Tight junction**	0.000307993	0.009424574
**Hypertrophic cardiomyopathy (HCM)**	0.000451195	0.009607269
**Dilated cardiomyopathy (DCM)**	0.000504977	0.009607269
**Bacterial invasion of epithelial cells**	0.000652441	0.009607269
**Shigellosis**	0.000676415	0.009607269
**Rap1 signaling pathway**	0.000691026	0.009607269
**Hippo signaling pathway -fly**	0.000752568	0.009607269
**Longevity regulating pathway - multiple species**	0.000753511	0.009607269
**Pathogenic Escherichia coli infection**	0.000894494	0.010527506
**Salmonella infection**	0.001022641	0.011176005
**Oxytocin signaling pathway**	0.001164876	0.011881735
**Vibrio cholerae infection**	0.001329054	0.012709079
**Phagosome**	0.001811521	0.015272017
**Gastric acid secretion**	0.001846737	0.015272017
**Viral myocarditis**	0.001896525	0.015272017
**Apoptosis**	0.002125888	0.015488613
**Hippo signaling pathway**	0.002125888	0.015488613
**Influenza A**	0.002311628	0.015916277
**Leishmaniasis**	0.002392643	0.015916277
**Hepatocellular carcinoma**	0.002720707	0.017344507
**Focal adhesion**	0.002984181	0.018263188
**Phototransduction - fly**	0.003129427	0.018415474
**Circadian rhythm - fly**	0.003448928	0.019543925
**Protein processing in endoplasmic reticulum**	0.003988674	0.021795254
**Fluid shear stress and atherosclerosis**	0.004987344	0.026312539
**Adherens junction**	0.007014203	0.035772435
**Proteoglycans in cancer**	0.009385437	0.046321673
**Thyroid hormone signaling pathway**	0.01014812	0.048520699
**Ether lipid metabolism**	0.01192332	0.054650682
**alpha-Linolenic acid metabolism**	0.01244013	0.054650682
**Endocytosis**	0.01250179	0.054650682
**Small cell lung cancer**	0.01741251	0.074003167

**Arachidonic acid metabolism**	0.02060517	0.085205162
**Valine, leucine and isoleucine biosynthesis**	0.02687528	0.105433791
**Neomycin, kanamycin and gentamicin biosynthesis**	0.02687528	0.105433791
**Chemical carcinogenesis**	0.03199252	0.121976955
**Linoleic acid metabolism**	0.03348387	0.121976955
**Jak-STAT signaling pathway**	0.03348387	0.121976955
**Ras signaling pathway**	0.03884064	0.137295072
**NF-kappa B signaling pathway**	0.03948355	0.137295072
**Thermogenesis**	0.04137684	0.140681256
**Toxoplasmosis**	0.04229931	0.140691183
**Intestinal immune network for IgA production**	0.04439645	0.143600554
**Fat digestion and absorption**	0.04585796	0.143600554
**MAPK signaling pathway - fly**	0.04598972	0.143600554
**Steroid hormone biosynthesis**	0.04806191	0.147069445
**The significant enrichment pathways in profile 5**		
**Pathway**	**Pvalue**	**Qvalue**
**Leukocyte transendothelial migration**	3.46E-06	0.000169401
**Platelet activation**	1.56E-05	0.000341456
**Influenza A**	2.09E-05	0.000341456
**Regulation of actin cytoskeleton**	4.85E-05	0.000485135
**Tight junction**	4.95E-05	0.000485135
**Vibrio cholerae infection**	8.37E-05	0.000518539
**Arrhythmogenic right ventricular cardiomyopathy (ARVC)**	8.37E-05	0.000518539
**Focal adhesion**	8.47E-05	0.000518539
**Gastric acid secretion**	0.000107527	0.000585426
**Phototransduction - fly**	0.000161486	0.000728913
**Bacterial invasion of epithelial cells**	0.000177585	0.000728913
**Shigellosis**	0.000181786	0.000728913
**Hippo signaling pathway -fly**	0.000194822	0.000728913
**Pathogenic Escherichia coli infection**	0.00021804	0.000728913
**Fluid shear stress and atherosclerosis**	0.0002329	0.000728913
**Salmonella infection**	0.000238013	0.000728913
**Oxytocin signaling pathway**	0.000259281	0.000747338
**Adherens junction**	0.000305904	0.000832738
**Viral myocarditis**	0.000358315	0.000902581
**Apoptosis**	0.00038682	0.000902581
**Hippo signaling pathway**	0.00038682	0.000902581
**Hepatocellular carcinoma**	0.000456897	0.001017634
**Rap1 signaling pathway**	0.000517397	0.00110228
**Phagosome**	0.000909211	0.001856305
**Hypertrophic cardiomyopathy (HCM)**	0.000978693	0.001918239
**Dilated cardiomyopathy (DCM)**	0.001036943	0.001954239
**Proteoglycans in cancer**	0.001082222	0.001964033

**Thyroid hormone signaling pathway**	0.001144762	0.002003334
**Thermogenesis**	0.001383947	0.002338393
**Cysteine and methionine metabolism**	0.003035297	0.004957652
