# Supplementary material for: Transcriptome Analysis on Hepatopancreas Reveals the Metabolic Dysregulation Caused by Vibrio parahaemolyticus Infection in Litopenaeus vannamei
Source: Biology (Basel). 2023 Mar 9;12(3):417. doi: 10.3390/biology12030417 (PMC10044748; doi:10.3390/biology12030417)
Supplement: Supplementary file 1 [file biology-12-00417-s001.zip › Table S4 Pearson correlation coefficient analysis between the results of RNA seq and RT-qPCR for twelve DEGs.pdf]

Table S4 Pearson correlation coefficient analysis between the results of RNA seq and RT-qPCR for twelve DEGs.

DEGs	Time post infection(hpi)	RT-qPCR	RNA-seq (FPKM)	Pearson Correlation Coefficient
**HK**	0	26.55	26.82	0.927736597
	6	77.70	84.38	
	12	197.59	117.48	
**FBA**	0	70.41	69.83	0.995717927
	6	73.90	123.02	
	12	477.10	555.32	
**LDH**	0	23.41	31.76	0.998805988
	6	33.12	49.48	
	12	143.07	165.82	
**PCK**	0	31.69	48.93	0.974376421
	6	48.79	114.35	
	12	89.10	180.34	
**G6PDH**	0	7.26	31.53	0.965740106
	6	6.48	44.49	
	12	18.96	91.53	
**PHGDH**	0	3.09	59.50	0.999258435
	6	2.15	49.79	
	12	13.95	304.64	
**SHMT**	0	10.53	45.61	0.984959443
	6	13.85	70.03	
	12	40.16	132.48	
**MTHFR**	0	31.95	24.07	0.998668024
	6	21.85	25.74	
	12	231.84	185.59	
**VEGFA**	0	2.70	4.44	0.988215468
	6	13.44	18.73	
	12	59.60	47.42	
**RTK**	0	47.96	25.95	0.983020625
	6	46.94	35.64	
	12	134.26	78.44	
**IκBα**	0	19.46	29.13	0.994929088
	6	27.19	43.33	
	12	54.66	74.46	
**Bcl-XL**	0	4.44	10.70	0.84134401
	6	5.98	20.01	
	12	11.47	23.47	
